# The Pathological Society

**Published:** 1897-02-06

**Authors:** 


### THE PATHOLOGICAL SOCIETY.

At the meeting held on February 2nd, while discussing certain cases of hæmorrhagic diphtheria related by Mr. Stephens and Mr. Purfitt, Dr. Kanthack drew attention to the waste of pathological material which occurred in many post-mortem rooms in this country. If such a complete pathological examination were made of the products of the post-mortem rooms as ought to be made, it would be found that in most of the fatal cases of diphtheria death was not due to diphtheria but to other intercurrent organisms. He thought, moreover, that if careful examination were made, it would in many cases be found that the growth of the diphtheria bacillus was not so purely local as had often been stated. Diphtheria bacilli certainly invaded the tissues.

Dr. A. E. Garrod related a case of pneumococcus endocarditis of a two-cusped aortic valve. There was suppuration in the middle ear; pus from the tympanum contained the pneumococcus, streptococci, and staphylococci; and cultures from the heart blood showed pneumococci. The patient died from pneumonia, the starting point of the infection evidently being the middle ear. A point of interest in the case was the apparent influence of an abnormality in the aortic valve in laying it open to the attack of an infective disease, for the micro-organisms must have passed through the right side of the heart, all the valves of which were healthy, and also through the mitral, and only set up endocarditis on arriving at the abnormal aortic valves, the congenital malformation of which seemed to have acted as a predisposing cause of the infective endocarditis by which they were ultimately attacked.

In regard to this point, Dr. Rolleston suggested that the malformation itself might be due to an endocarditis during fœtal life, in which case it would only be a matter of infective endocarditis attacking a valve which had already been diseased, not an uncommon event. Dr. Garrod, however, pointed out that each valve had its corpus aurantii placed in the centre of the curtain, which would hardly have been the case if they had been deformed by preceding inflammation; and Mr. Shattock thought that if the condition had been due to fœtal endocarditis there would have been stenosis, which was not the case.

A communication was read by Dr. Hector Mackenzie and Mr. W. Edmunds on the persistence of the thymus in Graves's disease. The fact seemed to be generally admitted. Dr. Bradford said that he had examined four cases of Graves's disease after death, and in all of them there was persistence of the thymus; but then the same was true in other conditions, such as Addison's disease and lymphadenoma. In regard to thymus feeding, Dr. Abrahams referred to certain authorities by whom it was maintained that this was useful in the non-exophthalmic cases, but not in Graves's disease.

